# *In Vitro* Microvessel Growth and Remodeling within a Three-dimensional Microfluidic Environment

**DOI:** 10.1007/s12195-013-0315-6

**Published:** 2013-12-03

**Authors:** Young K. Park, Ting-Yuan. Tu, Sei Hien Lim, Ivan J. M. Clement, Se Y. Yang, Roger D. Kamm

**Affiliations:** 1 Biosystems & Micromechanics IRG, Singapore-MIT Alliance for Research and Technology Center, Singapore 117543; 2 Department of Biological Engineering, Massachusetts Institute of Technology, Cambridge, MA 02139, USA; 3 Computational Biology Programme, Department of Biological Sciences, National University of Singapore, Singapore 119077; 4 Department of Mechanical Engineering, Massachusetts Institute of Technology, Cambridge, MA 02139, USA

**Keywords:** 3D microfluidic platform, Microvascular network formation, 3D gel scaffolds, Collagen gel, Fibrin gel

## Abstract

This paper presents *in vitro* microvascular network formation within 3D gel scaffolds made from different concentrations of type-I collagen, fibrin, or a mixture of collagen and fibrin, using a simple microfluidic platform. Initially, microvascular network formation of human umbilical vein endothelial cells was examined using live time-lapse confocal microscopy every 90 min from 3 h to 12 h after seeding within three different concentrations of collagen gel scaffolds. Among the three conditions of collagen gel scaffolds (2.0 mg/ml, 2.5 mg/ml, and 3.0 mg/ml), the number of skeleton within collagen gel scaffolds was consistently the highest (3.0 mg/ml), followed by those of collagen gel scaffolds (2.5 mg/ml and 2.0 mg/ml). Results demonstrated that concentration of collagen gel scaffolds, which influences matrix stiffness and ligand density, may affect microvascular network formation during the early stages of vasculogenesis. In addition, the maturation of microvascular networks in monoculture under different gel compositions within gel scaffolds (2.5 mg/ml) was examined for 7 d using live confocal microscopy. It was confirmed that pure fibrin gel scaffolds are preferable to collagen gel or collagen/fibrin combinations, significantly reducing matrix retractions during maturation of microvascular networks for 7 d. Finally, early steps in the maturation process of microvascular networks for 14 d were characterized by demonstrating sequential steps of branching, expanding, remodeling, pruning, and clear delineation of lumens within fibrin gel scaffolds. Our findings demonstrate an *in vitro* model for generating mature microvascular networks within 3D microfluidic fibrin gel scaffolds (2.5 mg/ml), and furthermore suggest the importance of gel concentration and composition in promoting the maturation of microvascular networks.

## INTRODUCTION

New blood vessels are formed by vasculogenesis and angiogenesis.^1^ Vasculogenesis is defined as the differentiation of precursor cells into endothelial cells (ECs), followed by *de novo* formation of primitive blood vessels. Angiogenesis, meanwhile, is defined as the sprouting of new blood vessels from pre-existing ones, followed by the growth of new capillaries.^1,2^ Vasculogenesis is relatively easy to reproduce *in vitro* because the early stages of vasculogenesis are accomplished with ECs as a single cell-type.^3^ Well-established *in vitro* systems employing phase-contrast or fluorescence microscopy^4,5^ have been used to examine the geometric properties of microvascular network formation during vasculogenesis. Previous studies on vasculogenesis were based on examining population averages at a fixed end point rather than the dynamic behaviors of the ECs.^4,5^ Recently, Parsa *et al.*^6^ developed a promising method for tracking vasculogenesis of primary ECs on Matrigel using time-lapse live-cell fluorescence microscopy. They presented a quantitative analysis of the dynamic behavior of ECs during the early stages of vasculogenesis at both single-cell and population levels. However, their results were not obtained within 3D extracellular matrix (ECM) environments using live confocal microscopy.

Recent studies^7–10^ have demonstrated the feasibility of growing perfusable microvascular networks *in vitro.* Such models hold enormous potential for the formation of stable vascular networks in engineered tissues. Considerable efforts were focused on creating *in vitro* models that generate several features of *in vivo* vascular microenvironment with fine spatial and temporal resolution.^7–11^ Based on an *in vitro* 3D angiogenesis or vasculogenesis model using a co-culture system of ECs with fibroblasts, Yeon *et al.*^10^ reported that perfusable tubular capillaries of various dimensions can be formed within microfluidic gel scaffolds (GSs) depending on the ratio of fibrin to collagen in the mixture. In a subsequent study from the same group,^12^ vessel formation by both angiogenesis and vasculogenesis was achieved by co-culture with fibroblasts, with the vessels remaining viable up to 7 d beyond the initial perfusion. The results of these studies offer insights for spontaneously generating accessible and perfusable blood vessels, including sensitivity to a range of experimental parameters and control over the size and shape of the lumen formed in their models.^13^ However, much remains to be learned about the various parameters that influence network formation and how these networks evolve and stabilize over time.

Previously, our laboratory developed microfluidic platforms for 3D cell culture and real-time imaging to investigate angiogenesis,^14^ cancer cell migration,^15^ interactions between different cell types found in the liver,^16^ and axon guidance.^17^ Furthermore, we investigated angiogenesis within 3D collagen gel scaffolds (CGSs) by culturing a monolayer of ECs on the channel walls under different experimental conditions.^14,18^ Based on our previous 3D systems, we developed a simple microfluidic platform to better understand the mechanisms underlying vasculogenesis, particularly during the initial steps of vasculogenesis as those observed *in vivo.* This versatile microfluidic platform allows simultaneous study of three discrete GSs containing different gel concentrations and/or compositions, which can be injected through separate gel ports. In addition, the small channel volume of this platform allows minimal consumption of valuable reagents, and offers flexible optical access at high resolution of 3D structures. Thus, this microfluidic platform advantageously offers a 3D extracellular matrix (ECM) environment, within engineered microfluidic GSs, to study *in vitro* microvessel remodeling during vasculogenesis.

We cultured human umbilical vein endothelial cells (HUVECs) inside 3D microfluidic GSs comprising three different concentrations of type-I collagen, collagen/fibrin mixtures, or fibrin; directly tracked the early process of vasculogenesis with live confocal microscopy; and qualitatively and quantitatively examined microvasculogenic behavior from 90 to 720 min after initial seeding within CGSs, and for 14 d within GSs of different collagen/fibrin compositions. Our results indicate that CGSs concentration, which determines both stiffness and ligand density, may affect microvessel formation during the early stages (first 12 h) of vasculogenesis. A direct comparison of microvasculogenic maturation within GSs of collagen and fibrin demonstrates that fibrin resists gel contraction, leading to long-term (14 d) stability for microvascular maturation. Therefore, the results demonstrate the influence of gel composition on the induction of early vasculogenesis and on early steps leading to the maturation of microvascular networks. Further, the results suggest that our microfluidic system may be useful in developing therapeutic strategies for the treatment of vascular dysfunction or tissue engineering.

## MATERIALS AND METHODS

### Fabrication and Characterization of a Simple Microfluidic Device

**FIGURE 1:**
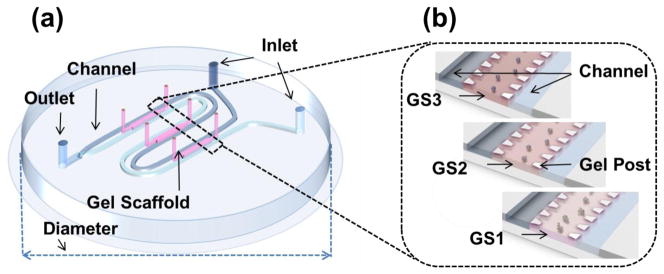
General features of a simple 3D microfluidic platform for tracking microvasculogenic behaviors within differently engineered microenvironments. (a) Schematic representation of the microfluidic device. The device with an overall diameter of 34 mm consists of two independent flow channels with inlet dimensions of 500 μm (width) × 150 μm (height) and three GSs with dimensions of 4.1 mm (length) × 1.3 mm (width) × 150 μm (height). The two independent flow channels merge at the outlet. (b) Schematic representation of enlarged 3D GSs. Three 3D GSs were created by injecting three different concentrations of type-I collagen gel or three different compositions of collagen and/or fibrin gel mixed with HUVECs through the gel ports. The 3D GSs were designated as GS1 (top), GS2 (middle), and GS3 (bottom) according to their position on the device and on the direction of inlet. Each GS contained 20 trapezoidal posts that were used to confine the gel to the region between the rows of posts.

A new microfluidic device (Fig. 1) was fabricated using polydimethylsiloxane (PDMS, Sylgard 184, Dow Chemical, MI) and soft lithography as previously described in standard microfluidic protocols.^14–18^ The device consists of two independent flow channels and three GSs, each containing 20 trapezoidal posts. The two independent flow channels merge at the outlet (Fig. 1a). The three GSs, GS1 (top), GS2 (middle), and GS3 (bottom) are designated according to their positions along the serpentine media channels and on the direction of flow (Fig. 1b). They were filled with three different concentrations of type-I collagen gel (BD Biosciences, MA, USA) or with three different compositions of collagen and/or fibrin gel mixed with HUVECs through the gel ports (Fig. 1b). Note that the system used in this study contained three regions, although the same configuration could be extended to include any desired number of gel regions depending on the application.

### Seeding of HUVECs within 3D CGSs

HUVECs were isolated using collagenase treatment, seeded on fibronectin-coated plates, and cultured in a medium containing Earle’s salts and fetal calf serum (Life Technologies, Grand Island, NY, USA) without growth factor supplementation, for 7 d at 37 °C in a 5% CO_2_ incubator.^19^ For our experiment, these HUVECs were cultured in an endothelial growth medium (EGM-2mv, Lonza, MD, USA) and used from passage 4 through passage 6. The confluence of HUVECs was monitored using phase-contrast microscopy.

Different concentrations of type-I collagen gel were used within GS1, GS2, and GS3 and selected on the basis of previous studies to maintain gel stability.^14–16^ We used EGM-2mv medium except vascular endothelial growth factor (VEGF; R&D Systems, Minneapolis, MN, USA) and supplemented it with 40ng/ml of VEGF (EGM-2mv-SV40). The volume of the seeded HUVECs (3 × 10^6^ cells/ml) stained with 5-chloromethylfluorescein diacetate (CMFDA, Cell Tracker^™^ Green; Invitrogen, Carlsbad, CA, USA) did not exceed 10% of the total GS mixture. The collagen gels were allowed to polymerize for 40–45 min at 37 °C in 5% CO_2_. Following polymerization, 300 μl of EGM-2mv-SV40 medium was injected through one port and subsequently incubated at 37 °C in 5% CO_2_ for observing initial vasculogenesis. HUVEC culture medium was replaced every 24 h.

### Quantitative Analysis of Microvasculogenic Behaviors within 3D CGSs

The early stage of capillary network formation was examined under a phase contrast (Olympus CKX41) or confocal microscope (Olympus Fluoview FV1000) from 90 min to 12 h after initial seeding of HUVECs within the three CGSs. For quantitative tracking of microvasculogenic behaviors, time-lapse mosaic imaging was conducted by examining three microscopic fields (100% of the total area of each CGS). Using 10X UPLFLN (NA: 0.30), Z-stack images were generated by scanning every 3.5 μm of CGSs (150 μm). Time-lapse mosaic images of a Z stack were subsequently processed using FV10-ASW version 2.0 (Fluoview, Olympus), IMARIS 6.4 software for 3D visualization and analysis (IMARIS, Bitplane Scientific Software), and/or Image J software. All time-lapse images for confocal microscopy were processed using the maximum intensity projection (MIP) of the Z-stacks (Supplementary Material (SM)). To distinguish the cells from the background, contrast adjustment and thresholding were carried out on the MIPs. The visual quality of the images was enhanced by removing residual background noise. Processed images were skeletonized using an Image J plugin^20^ and implementing a thinning algorithm for 2D/3D skeletonization.^21^ The skeletonized representation of the vascular network was mathematically formalized as an undirected graph consisting of nodes and edges. In this view, the number of skeletons was defined to be the number of connected components in graph. The spine of each skeleton was computed as follows. First, all the endpoints (nodes having a degree of one) in the skeleton were identified. The shortest paths between all possible pairs of endpoints were subsequently computed. The longest among all these shortest paths was defined as the spine of skeleton. After identifying the spine of the skeleton, any node on the spine that has a degree greater than three was labeled a junction, and any path that started from a junction but did not lie on the spine was counted as a branch. After identifying the spine of the skeleton, any node on the spine that has a degree greater than three was labeled a junction, and any path that started from a junction but did not lie on the spine was counted as a branch. The number of skeletons, branches, and junctions were counted from the skeletonized MIPs using the Image J plugin and plotted as a normalized number against the maximum value of skeletons, branches, and junctions from eight to ten images within five to six different devices at three different times (N = 3) under different experimental conditions. The normalized value is defined as the value divided by the maximum value for any given data set. Statistical analyses were performed to compare these metrics across three different GSs. Statistical analyses were performed to compare these metrics across three different GSs or two different time intervals. Student’s t-test was used to perform all statistical comparisons. Statistical significance was assumed whenever p < 0.05. All data are reported as mean ± SD or plotted as median with upper (75%) and lower (25%) quartiles.

### Characterization of Microvasculogenic Maturation within 3D Fibrin Gel Scaffolds (FGSs)

To promote stabilization of the microvascular networks within CGSs (2.5 mg/ml of type-I collagen gel) for the long term, HUVECs were cultured in EGM-2mv-SV40 medium for 12 h until confirming fully microvascularized networks across CGSs from the top to the bottom channel. After 12 h, we added GM6001 (Millipore, Jersey City, NJ, USA) (12.5 μM) – a broad-spectrum matrix metalloproteinase (MMP) inhibitor – not only to prevent spontaneous retraction of collagen gels from the active growth of the HUVECs, but also to generate stable microvascular networks within the CGSs.^22^ At days 2 and 6 after the initial seeding, stabilized microvascular networks and lumen formation within the CGSs were qualitatively examined. Subsequently, we comparatively examined spontaneous retractions of GSs and maturation of microvascular networks within GS1, GS2, and GS3 with collagen gel (2.5 mg/ml), collagen gel mixed with fibrin gel (1:1 mixture), and fibrin gel (2.5 mg/ml), respectively, at days 2 and 7. The preparation of different gel compositions was followed by the experimental protocol described in the SM. To evaluate the degree of retraction of the three different compositions of GSs at days 2 and 7, we assigned quantitative grades for them using the protocol in SM. Based on the optimal gel composition within GSs on the comparative results for the degrees of retraction, we characterized the maturation of microvascular networks within FGSs over the course of 14 days using the protocol previously described.

### Immunohistochemistry

After initial seeding, we examined the mature microvascular networks within FGS samples using immunohistochemistry-based fixed staining. FGS samples were fixed with 4% paraformaldehyde (Sigma-Aldrich, St. Louis, MO, USA) for 15 min at room temperature and washed with phosphate-buffered saline (PBS). We used 0.1% triton X-100 (Sigma-Aldrich, St. Louis, MO, USA) to permeabilize the microvascular networks at room temperature. FGS samples were blocked with 0.5% bovine serum albumin (BSA; Sigma-Aldrich, St. Louis, MO, USA) in blocking buffer for 2 h at room temperature. FGS samples were then incubated at 4 °C overnight with a 1:1000 dilution of VE-cadherin primary antibodies (Enzo Life Sciences, Farmingdale, NY, USA). They were subsequently incubated with a 1:100 dilution of Alexa Fluor 488-conjugated goat anti-rabbit secondary antibodies (Molecular Probes, Eugene, OR, USA) for 2 h at room temperature. In addition, the FGS samples were counterstained with Hoechst 33342 (10 μg/ml; Invitrogen, Carlsbad, CA, USA) for 30 min at room temperature. Fluorescence was detected using confocal microscopy.

## RESULTS AND DISCUSSION

### Formation of Microvascular Networks within 3D CGSs

**FIGURE 2:**
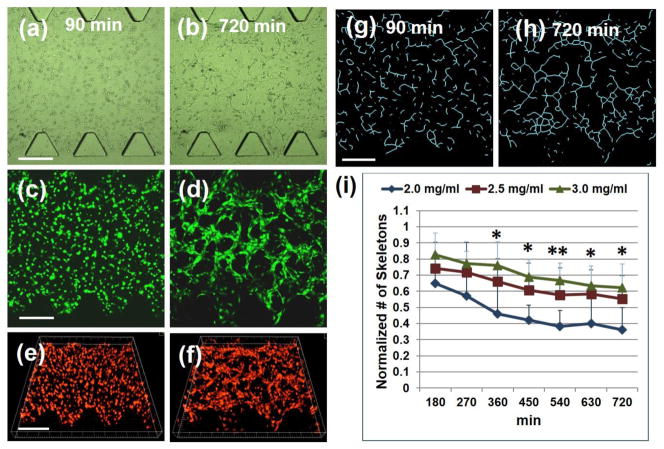
Microvasculogenic behaviors within 3D CGSs (2.5 mg/ml) from 90 min to 720 min after initial seeding of HUVECs. (a)–(b) Phase contrast microscopic images at 90 min and 720 min (c)–(d) Fluorescence detection images using confocal microscope at 90 min and 720 min. (e)–(f) Confocal microscopic images processed using IMARIS 6.4 software at 90 min and 720 min. (g)–(h) Representative skeletonized images at 90 min and 720 min. (i) Kinetics of microvasculogenic behaviors within 3D CGSs from 90 min to 720 min. Collagen concentrations were 2.0 mg/ml (CGS1), 2.5 mg/ml (CGS2), and 3.0 mg/ml (CGS3), respectively. Quantitative analysis was conducted by examining skeleton formation from MIP images at the indicated time points. Comparative data among CGSs are plotted as the normalized skeleton numbers from eight to ten images within five different devices at three different times (N = 3). The normalized value is defined as the value divided by the maximum value for any given data set. Results were compared using the Student’s t test. Data are plotted as mean ± SD (* p < 0.05; ** p < 0.009), vs. skeleton count in CGS2 or CGS3. The maximum value of skeletons for the normalization procedure was 404. Scale bar, 300 μm.

The formation of microvascular networks of ECs depends on cell seeding density, concentration of VEGF, collagen gel concentration in CGSs, and incubation time after initial seeding. To create *in vitro* optimal conditions for forming capillary networks of ECs, each GS with different concentrations of type-I collagen gel was seeded with 3 × 10^6^ ECs/ml in standard EGM-2 EC media with VEGF supplement (0, 20, 40, 60 or 80 ng/ml) from 2 h to D6. Collagen gel concentrations of 2.0 mg/ml, 2.5 mg/ml, and 3.0 mg/ml were chosen for study within CGS1, CGS2, and CGS3, respectively, based on previous experiments in similar microfluidic systems.^14–16^ Within these ranges, pilot experiments using phase contrast microscopy identified optimal experimental conditions of 3 × 10^6^ ECs/ml, 40 ng/ml VEGF, and 2.0 mg/ml and 2.5 mg/ml of type-I collagen. We then qualitatively examined microvasculogenic behaviors within 3D CGSs (2.5 mg/ml) under the experimental conditions of 3 × 10^6^ HUVECs/ml and 40 ng/ml of VEGF from 90 min to 720 min after initial HUVEC seeding using phase contrast microscopy (Figs. 2a and 2b), fluorescence microscopy (Figs. 2c and 2d), and confocal microscopy (Figs. 2e and 2f). In addition, we extracted skeletonized images at 90 and 720 min (Figs. 2g and 2h), recognizing that, due to the 3D nature of the structures, these images would underestimate the number of independent, non-connected segments. At 90 min, the cells were still in the process of extending and forming connections with their neighbors, but by 720 min, most of the cells were interconnected, forming nearly complete microvascular networks. These results are consistent with those of other groups studying *in vitro* vascular network formation.^4,6^ Such trends result from the elongation and migration of individual cells, ultimately forming adhesive contacts with their neighbors.^3,4^

### Kinetics of Microvascular Morphogenesis under Different Gel Concentrations

We also examined the extent of microvascular network formation at different gel concentrations during the early stages of vasculogenesis. We analyzed early vasculogenesis based on the modified model of skeleton pruning ^21^ every 90 min from 90 to 720 min after initial seeding (Fig. 2i). Among the three conditions tested (2.0 mg/ml, 2.5 mg/ml, and 3.0 mg/ml), the number of apparent skeletons fell with increasing time, and was consistently highest at a collagen concentration of 3.0 mg/ml, followed by 2.5 mg/ml and 2.0 mg/ml, respectively. Statistical significance of these trends (p < 0.05) emerged at the later time points. Live confocal images at the time points indicated on the graph of Fig. 2i and skeletonized images at 540 min (Fig. S1) clearly show significant differences between CGS1 and CGS3. These results demonstrate that collagen gel concentration, which influences collagen gel stiffness and ligand concentration, affects microvascular network formation during the early stages of vasculogenesis. In addition, these results, as a whole, suggest that microvascular network formation is facilitated at lower gel concentrations especially during the first 720 min of vasculogenesis. Cell elongation and migration occur in order that networks might form, and the lower gel concentration might well promote these phenomena.

It is well established that other than responding to biochemical signals, ECs also sense their mechanical environment and respond accordingly.^23^ Various groups have demonstrated the effect of matrix stiffness on vascular network formation^24–26^ and developed mathematical models to capture these matrix-dependent behaviors.^27,28^ In particular, it has been shown that vascular networks are more likely to form if the matrix stiffness is low.^27^ Recent studies have examined the effects of matrix concentration and stiffness on capillary morphogenesis using pure ECM components with different compositions and stiffnesses. These studies reported that the surrounding matrix modulates the degree of new vessel formation and suggested an inverse relationship between matrix stiffness and the degree of vascular network formation. Most recently, Rao *et al.*^29^ demonstrated that the matrix composition regulates vasculogenesis in 3D collagen/fibrin hydrogels and suggested that this is because of the mechanical properties of these gels. However, experimental treatments of the mechanical properties also modulated the ECM architecture, such as changes in mass transport, ligand density, and other parameters that affect vasculogenesis.^29–33^ Decoupling the individual effects of ECM architecture, such as matrix stiffness, chemistry, porosity, and elasticity, on vasculogenesis in 3D gels remains a challenge. We confirmed that collagen concentrations of 2.0 mg/ml and 2.5 mg/ml are more conducive to microvascular network formation than a collagen concentration of 3.0 mg/ml. Furthermore, we chose to use 2.5 mg/ml for all remaining experiments as a compromise between optimal network formation and reduced matrix degradation and contraction.

### Stabilization of Microvascular Networks

To stabilize the formed microvascular networks, GM6001, a broad-spectrum MMP inhibitor, was used to prevent matrix degradation. Initially, the microvascular networks were induced by the addition of 40 ng/ml VEGF in combination with 12.5 μM GM6001 to the 3D CGS (2.5 mg/ml) for 6 d after initial seeding. Under these conditions, most of the CGSs still exhibited gel retraction that began near the gel posts after 2 d that impeded further microvascular network formation, and which continued to increase through day 6 (Fig. S2). A tendency similar to that reported by Yeon *et al.*^10^ was observed, i.e., gel retraction was followed by detachment from the PDMS surface using gel mixtures that included high concentrations of type-I collagen or collagen without fibrin. Our results are consistent with their results and suggest that the cellular contraction within the type-I collagen scaffolds results in detachment of the matrix from its surroundings and destroys the intended geometry.^34^ We anticipate potential roles of type-1 collagen in supporting vasculogenesis *in vitro*, with the degree of vessel-like structure formation depending on the composition of the mixture in the GSs.^29^ However, the mechanisms by which type-I collagen regulates vascular morphogenesis are still unclear, despite considerable research on type-I collagen as a potential promorphogenic molecule in vasculogenesis.^29,34^

**FIGURE 3:**
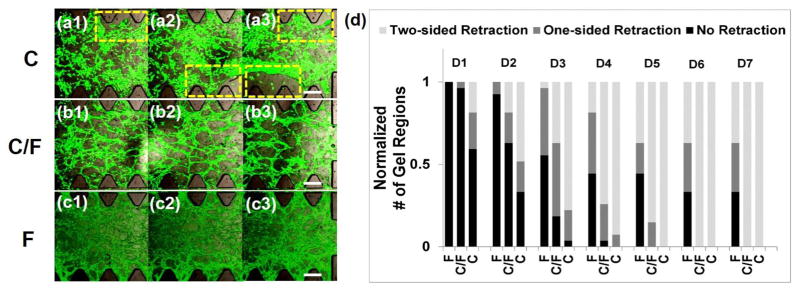
Evaluation of spontaneous retraction of microvascular networks formation. Live confocal images of microvascular networks within GS1 [(a1)–(a3)], GS2 [(b1)–(b3)], and GS3 [(c1)–(c3)] at day 2. The GSs composed of collagen (2.5 mg/ml), collagen mixed with fibrin (1:1), and fibrin (2.5 mg/ml) are designated as C, C/F, and F, respectively. Retraction areas are indicated by dashed yellow lines. Scale bars, 300 μm. (d) Comparative scoring for spontaneous retractions within GS1, GS2, and GS3 from days 1 (D1)–7 (D7). X axis represents three different GSs with two-sided retraction, one-sided retraction, and/or no retraction and Y axis represents the normalized number of total gel regions within GSs from days 1–7. The normalized number of total gel regions was obtained by normalizing the number of gel regions that either had two-sided retraction, one-sided retraction or no retraction against the summation of these three numbers for each GS composition. The summation of total gel regions for each GS composition is 27. Retraction is defined as the areas with a retraction distance >250 μm from the border between the channel and the GSs. The grades for the degree of retraction are: 0 = two-sided retraction, 0.5 = one-sided retraction, and 1 = no retraction. Results in 6 devices at 3 times (N = 3) are compared.

To reduce the tendency for gel retraction within CGSs, we tried using different gel compositions and mixing collagen with fibrin. This strategy was based on previous microfluidic studies on the formation of ECs networks in fibrin/collagen mixtures.^10^ We compared the degree of spontaneous retraction with collagen (2.5 mg/ml), collagen mixed with fibrin (1:1 mixture), and fibrin alone (2.5 mg/ml), in GS1, GS2, and GS3, respectively, for 7 d. Representative live confocal images of microvascular networks within whole areas of GS1, GS2, and GS3 at day 2 show no contraction in GS2 (Figs. 3b1–3b3) or GS3 (Figs. 3c1–3c3), but clear retraction on one side (Figs. 3a1 and 3a2) or both (Fig. 3a3) in the vicinity of the posts within GS1 (highlighted by the dashed yellow lines). To convert these observations into a quantitative metric, we defined the retraction areas as those with a retraction distance >250 μm from the border line between the channel and GSs. GSs with two-sided, one-sided, and no retraction were scored as 0, 0.5, and 1, respectively. Fig. 3d shows comparative quantification data on the degree of retraction within the three different GSs. Two-sided retractions for CGSs, mixed gel scaffolds (MGSs) of collagen and fibrin, and FGSs are observed on days 1, 2, and 3, respectively (Fig. 3d). In addition, CGSs and MGSs had no non-retracting regions beyond days 4 and 5, respectively. However, we observed that some FGSs could be maintained without retractions until day 7. In terms of structural geometry without gel retraction within 3D FGSs, our results showed the same tendency as those of Carrion *et al.*,^11^ who used ECs embedded in 3D fibrin gels without gel retraction from the surface of PDMS to stimulate a morphogenetic process similar to vasculogenesis. One explanation for the desirable properties of fibrin gels may be their ability to withstand large strains without generating high stress. Compared with collagen gels, fibrin gels are considerably more highly extensible and can withstand much greater strains before rupture.^35^ This may allow some contraction of the vascular network without pulling the gel away from the sides, posts, or walls.

While we did not conduct comparative rheological or morphological studies of the two gels, mechanical properties and porosity of our gels may be gleaned from the literature. It has been shown that, at comparable concentrations, pure fibrin gels tend to have smaller pore sizes than collagen, ranging from 0.6 to 7.4 microns, respectively, at a concentration of 2.0 mg/ml,^36^ Pore size could affect transport of signaling molecules, but these sizes for a highly hydrated gel should have little impact on diffusion coefficients. There appears to be more variability in the reported values of elastic modulus, however, with fibrin being either about four times stiffer ^36^ (higher G′) or ~10 time less stiff ^37^ (lower G′) than collagen. What seems clear, however, is that fibrin, under high levels of strain as occur in the present experiments, is much more compliant than collagen,^37^ and that fibrin can attain higher levels of strain than collagen before fracturing.^37^ In addition, there are well-documented differences in the integrins to which collagen and fibrin bind, as well as differences in the matrix metalloproteinases (MMPs) that they are degraded by.^38^ Given these findings, we speculate that fibrin gels can undergo larger strains but exert smaller forces at the boundaries. At the same time, it is possible that the amount of cell contraction, as well as the ability of the cells to degrade and remodel the matrix, could be different in fibrin than in collagen. Taken together, several factors could be responsible for the improved properties of fibrin as a matrix for microvascular networks, however, the present experiments are unable to identify which of these is most important.

**FIGURE 4:**
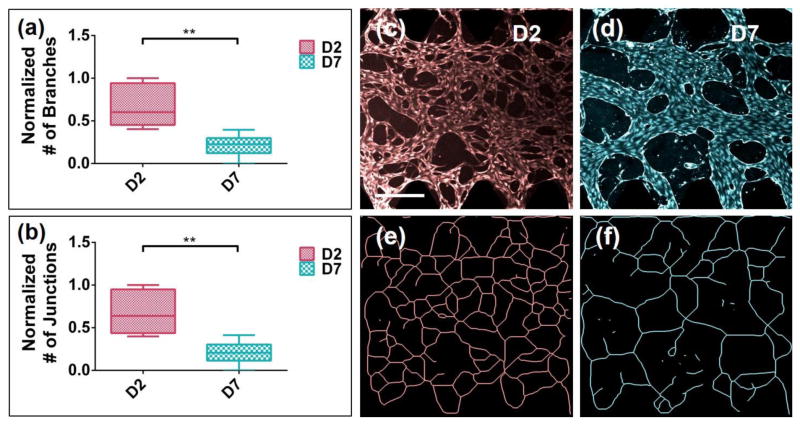
Quantification of microvascular network formation within 3D FGSs at days 2 and 7. (a)–(b) Quantification of microvascular network formation within 3D FGSs. Box and whisker plots show the decreasing normalized numbers of branches and junctions between days 2 (D2) and 7 (D7), respectively. The normalized value is defined as the value divided by the maximum value for any given data set. The maximum values of branches and junctions were 365 and 225, respectively. Results are within six different devices at three different times (N = 3). Data are plotted as median with upper (75%) and lower (25%) quartiles. Error bars represent max and min values (** p < 0.003). (c)–(d) Representative confocal microscopic images at days 2 (D2) and 7 (D7), respectively. Scale bars, 300 μm. (e)–(f) Corresponding skeletonized images of (c)–(d).

Microvascular network formation was also influenced by the composition of GSs, as assessed by counting the number of branches and junctions at days 2 and 7 after initial EC seeding (Figs. 4a and 4b). The formation of both branches and junctions decreased between days 2 and 7 (p < 0.003). A pattern was observed exhibiting a tendency toward stabilization and further maturation of microvascular networks in fibrin. Live confocal images of microvascular networks are represented by fluorescence images (Figs. 4c and 4d) and corresponding skeleton-based metrics (Figs. 4e and 4f) at days 2 and 7, respectively. Our results showing initial microvascular network formation within FGSs at day 2 and maturation by day 7 provide some support for previous results from other groups on *in vitro* vascular network formation.^10,11^

It has been reported that ECs within 3D ECM, including type-I collagen or fibrin, form adhesions with surrounding ligands when exposed to growth factors during angiogenesis or vasculogenesis.^22,39^ Under these conditions, the growth factors are unable to act alone and trigger angiogenesis, and the ECs need to establish concurrent integrin-mediated adhesive interactions with matrix-bound ligands during neovascularization.^40,41^ Yeon *et al.*^10^ showed that ECs within gel mixtures lacking collagen could not form tip cells for angiogenesis or vacuoles that form the lumen and undergo regression after a few days. Compared to their observations of angiogenesis, we observed vasculogenesis directly from ECs embedded within CGSs, MGSs, and FGSs with different degrees of microvascular network formation, regardless of the presence of type-I collagen (Figs. 4a and 4b). Previously, it has been shown that lumen and tube formation by ECs in collagen matrices is regulated by X2P1 integrin–ECM signaling.^40^ In addition, fibrin provides specific adhesion sites for integrin receptors expressed on angiogenic endothelial cells, such as α_v_β_3_, α_v_β_5_, and α_5_β_1_.^41,42^ The structure of the fibrin-based matrix not only provides the necessary binding sites for EC integrins but also determines the rate and extent of the proteolytic degradation during ECs angiogenesis.^43^ Based on these results, we suggest that type-I collagen induces the initial angiogenesis in EC monolayers toward 3D GSs and that fibrin promotes GSs stability without gel retraction, in various integrin-dependent mechanisms, during angiogenesis or vasculogenesis. Taken together, we conclude that collagen and fibrin matrices support vasculogenesis *in vitro* in an integrin-dependent manner, but the degree of vessel-like structure formation is dependent on the mixture composition.^29^ However, the detailed mechanisms regulating type-I collagen or fibrin-based integrin–ECM signaling during vasculogenesis remain to be elucidated.

### Maturation of Microvascular Networks within FGSs

**FIGURE 5:**
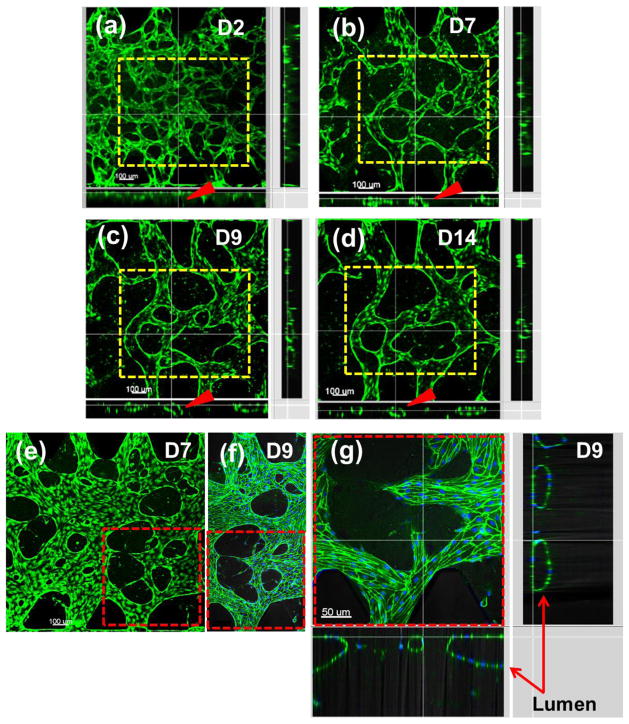
Characterization of mature microvascular networks within FGSs over 14 d. (a)–(d) Representative confocal images displaying the sequential steps of branching, expanding, remodeling, and pruning of microvascular networks in the area highlighted by the dashed yellow lines within FGSs at days 2 (D2), 7 (D7), 9 (D9), and 14 (D14), respectively. Red arrows indicate lumen structures. Scale bars, 100 μm. (e)–(f) Live and immunohistochemistry-based confocal images of mature microvascular networks at D7 and D9, respectively. Immunohistochemistry-based staining with VE-cadherin and Hoechst 33342 are shown in green and blue, respectively. Areas highlighted by the dashed red lines represent the remodeling steps of the maturation of microvascular networks at D7 and D9, respectively. Scale bar, 100 μm. (g) Cross-sectional image of the area highlighted by the dashed red line in (f) by XZ and YZ axis at D9. Immunohistochemistry-based staining with VE-cadherin and Hoechst 33342 are visualized in green and blue, respectively. Red arrows represent lumen formation. Scale bar, 50 μm.

Based on the comparative retraction results (Figs. 4a and 4b), we used FGSs as the preferred 3D microenvironment to assess the maturation of microvascular networks during a 2-week experiment. In our experimental results, vascular maturation is characterized by the sequential steps of branching, expansion, remodeling, pruning, and lumen formation in the microvascular networks within the FGSs (Fig. 5). This process is evident in the sequential steps of maturation from days 2–14 (see the yellow boxed regions in Figs. 5a–5d), and even over a shorter 2-day period in another FGS at days 7 and 9 (see the red boxed regions in Figs. 5e and 5f, respectively). In addition, although lumen formation was not clearly observed at day 2 (Fig. 5a), it was clearly evident by days 7, 9, and 14 (see red arrows in Figs. 5b–5d). The lumen formation at day 9 is also very clearly seen in the cross-section (XZ and YZ planes) of the red-boxed region in Fig. 5f. Fig. 5g shows the confocal images prepared by immunohistochemistry-based staining with VE-cadherin (green) and Hoechst 33342 (blue). VE-cadherin (Figs. 5f and 5g), is essential for the formation and regulation of endothelial cell junctions, and also the modulation of the various cellular processes of endothelial cells, such as tube and lumen formation of blood vessels.^44^ Maturation of the vascular network involves optimal patterning of the network by branching, expanding, remodeling, and pruning to meet local demands within a proper 3D ECM environment. Engineered tissue requires blood vessels to grow and to remain viable within the 3D ECM. Therefore, initial efforts toward this purpose should be focused on successfully generating primitive vascular networks derived from ECs culturing within *in vitro* 3D matrix.

## CONCLUSIONS

This paper characterizes microvascular network formation using different concentrations of type-I collagen, fibrin, or a mixture of collagen and fibrin in a simple microfluidic platform. The experiments using type-I collagen show that microvascular network formation is favored at lower matrix concentrations, combining lower stiffness and increased permeability with lower ligand density. In a comparison of different matrix materials, fibrin gel was found to be preferable to collagen or mixed collagen-fibrin gels because of its reduced relative contraction, as observed over 7 d. We demonstrated the *in vitro* generation of mature microvascular networks within fibrin gels (2.5 mg/ml) and suggest that both gel composition and concentration influence the maturation of microvascular networks. The challenge now is to create both structurally and functionally mature microvascular networks *in vivo* to benefit regenerative medicine and treatment of various ischemic diseases or to destroy a pathological network completely to treat various angiogenic diseases including cancer.

## Supplementary Material

12195_2013_315_MOESM1_ESM
