# Supplementary material for: In Vitro Microvessel Growth and Remodeling within a Three-dimensional Microfluidic Environment
Source: Cell Mol Bioeng. Author manuscript; Available in PMC 2015 Mar 1. (PMC3960002; doi:10.1007/s12195-013-0315-6)
Supplement: 12195_2013_315_MOESM1_ESM [file NIHMS545808-supplement-12195_2013_315_MOESM1_ESM.docx]

**Electronic Supplementary Material**

***In Vitro* Microvessel Growth and Remodeling within a Three-dimensional Microfluidic Environment**

Young K. Park,^1,2^ Ting-Yuan. Tu,^1^ Sei Hien Lim,^1^ Ivan J. M. Clement,^3^ Se Y. Yang^4^ and Roger D. Kamm^1,2,4^

^1^Biosystems & Micromechanics IRG, Singapore-MIT Alliance for Research and Technology Center, Singapore 117543; ^2^Department of Biological Engineering, Massachusetts Institute of Technology, Cambridge, MA 02139, USA; ^3^Computational Biology Programme, Department of Biological Sciences, National University of Singapore, Singapore 119077; and ^4^Department of Mechanical Engineering, Massachusetts Institute of Technology, Cambridge, MA 02139, USA

**Materials and Methods**

*Preparation of Different Gel Compositions within 3D Gel Scaffolds.*

First, we prepared several vials (100 µl in each vial) of collagen gels (2.5 mg/ml) and maintained them in an ice box just before injecting into gel scaffolds (GSs). Next, we prepared fibrin gels very carefully due to their immediate polymerization at room temperature. The prepolymer solution (1–3 ml) containing bovine fibrinogen (2.5 mg/ml) (Sigma, St. Louis, MO. USA) and thrombin (50 U/ml) (2 µl) (Sigma, St. Louis, MO. USA) was separately prepared within an ice box. Subsequently, human umbilical vein endothelial cells (HUVECs) were suspended in the prepolymer solution (100–200 µl) as a mixing ratio of 1:10 within an ice box. The preparations of bovine fibrinogen (100 µl) including HUVECs and thrombin (2 µl) were mixed as fast as possible within an ice box. Finally, the produced preparations from collagen gel, mixed gels for collagen gels and fibrin gels, and fibrin gels were immediately embedded within three different GSs, before polymerization. After transferring them to humidity box, HUVECs-seeded GSs were allowed for polymerization during 40 min at incubator of 37 °C and 5% CO2.

*Evaluation of Retraction Degrees for Three Different Gel Scaffolds*

First, we defined 1/3 area of each GS as one unit for scoring retraction degrees from the time-lapse mosaic images using confocal microscopy. Next, we comparatively evaluated spontaneous retractions of GSs within GS1, GS2, and GS3, with collagen gel (2.5 mg/ml), collagen gel mixed with fibrin gel (1:1 mixture), and fibrin gel (2.5 mg/ml), respectively, an everyday for long term during 7 d. In order to convert these observations into a quantitative metric, we defined the retraction areas as those with a retraction distance > 250 µm from the border line between the channel and GSs. GSs with two-sided retraction, one-sided retraction, and no retraction were scored as 0, 0.5, and 1, respectively. Results within six different devices at three different times (N = 3) are compared using the evaluation of retraction degrees. We represented Y axis as the normalized number of total gel regions within GSs and X axis as three different GSs with two-sided retraction, one-sided retraction, and/or no retraction from days 1–7.

**Results and Discussion**

*MIP as a Tool for 3D Rendering of the Vasculature*

As previously known in the field of the visualization for 3D vessel volumetric data, MIP has become an acceptable tool for 3D rendering of the vasculature despite the shortcomings of the algorithm.^1^ Vessel segmentation algorithms are critical components of the analysis systems for circulatory blood vessels.^2^ Such segmentation algorithms are skeleton-based methods, which extract blood vessel centerline to use for analyzing and classifying the blood vessels.^2^ Recently, various thinning or skeletonization algorithms for 3D images have been developed to extract shape features for allowing simultaneous morphological and molecular analysis of thick tissue samples. These algorithms aid in overcoming the penetration limits of other microscopy modalities such as confocal microscopy and two-photon excitation microscopy. Many of the methods described and used in the 3D skeletonization have been developed as ImageJ plug-ins^3^ and can be freely downloaded from <http://bioweb>. cnb. csic.es/_iarganda /software.html.

In many circumstances, 3D image analysis can be implemented using a Volume Rendering method or as a MIP. Rendering with either technique consists of three steps: volume formation, classification, and image projection.^1^ However, there are many adjustable parameters, and thus, there is no standardization of volume rendering methods among vendors for most volume rendering methods. Generally, the parameters from one system do not only translate sufficiently to another, and image quality also varies among vendors.^1^

MIP is a widely used 3D rendering tool,^4,5^ and has been shown to be more accurate than surface rendering for evaluating the vasculature.^6,7^ However, correct interpretation of MIP images requires an understanding of the MIP algorithm and consequently, of the limitations of MIP. Most significant limitations include (a) the presence of other high-attenuation voxels may obscure evaluation of the vasculature and (b) the 3D relationships among the structures in the display are not visible.^8^ The first limitation is a most common problem when using vessel images for diagnostic purposes, such as evaluating atherosclerotic vessels.^9,10^ This limitation is usually overcome by slab editing of the volume for removing other high-attenuation voxels either from adjacent vessels or from bone.^11,12^ The second limitation is that the MIP display is a 2D representation that cannot depict accurately the actual 3D relationships of the vessels.^12,13^

There tends to be much less variability in MIP image reconstruction than in volume rendering because of the fewer parameters factored into the MIP algorithm. Most MIP methods use only windowing parameters considered as standardized measures that should be valid across different vendors’ systems. Nonetheless, it has been reported that differences in image quality are generated from reconstruction with different MIP algorithms.^14^ On the other hand, volume rendering enables a color display, which often improves the visualization of complex anatomy and 3D relationships.^15^ As a result, MIP may allow visualization of smaller branch vessels with less work than that required for volume rendering.^5^

*Kinetics of Microvascular Morphogenesis under Different Gel Concentrations*

**
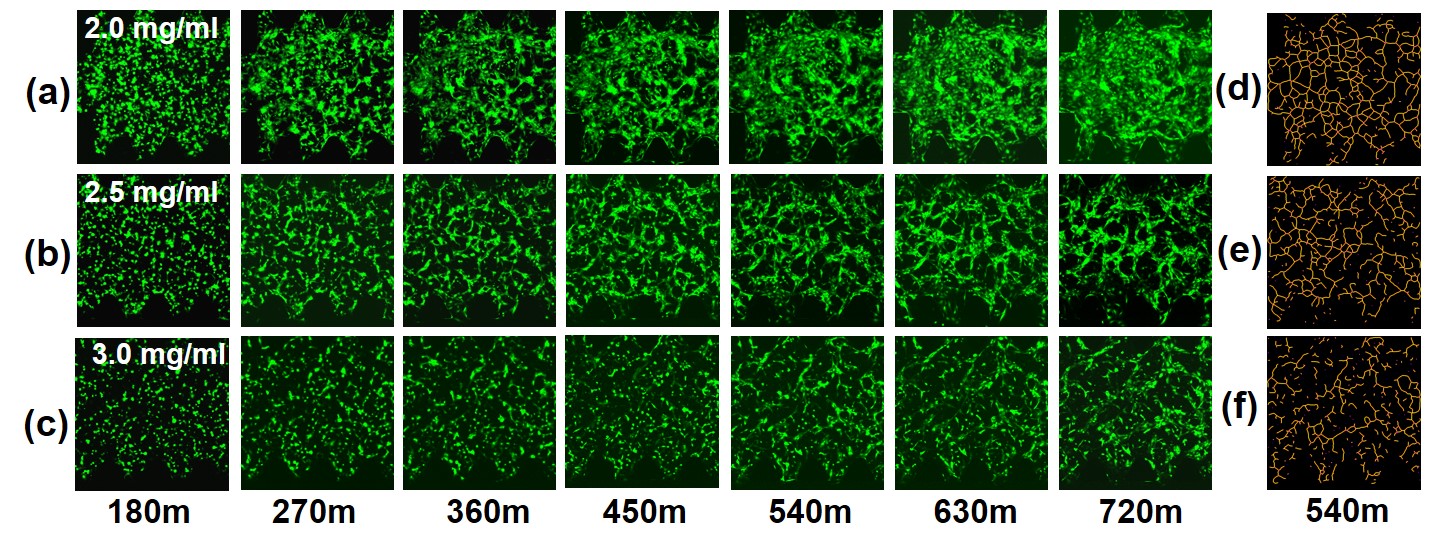
**

**FIGURE S1. Representative live confocal images for kinetics of microvasculogenic behaviors within 3D CGSs from 90 to 720 min after initial seeding of HUVECs. Collagen concentrations were 2.0 mg/ml (CGS1), 2.5 mg/ml (CGS2), and 3.0 mg/ml (CGS3), respectively. (a)–(c) Representative live confocal images shown from three CGSs at the time points indicated on the graph of Fig. 2i. (d)–(f) Skeletonized images shown from three CGSs at 540 min. The experimental conditions and analysis were same as those of Fig. 2i.**

*Stabilization of Microvascular Networks*

Stabilization of microvascular networks under different gel compositions within 3D gel scaffolds. To stabilize the formed microvascular networks, we used VEGF to stimulate microvascular network formation, and GM6001, a broad-spectrum MMP inhibitor, to prevent matrix degradation. Initially, we stabilized the microvascular networks by adding VEGF (40 ng/ml) and GM6001 (12.5 μM) to the 3D collagen cultures (2.5 mg/ml) for 6 d after initial seeding (Fig. S2). However, we excluded any molecules that govern cell-cell interactions since we have not used coculture of ECs with mural cells to develop microvascular networks *in vitro*. Under these conditions, most of the collagen gel scaffolds (CGSs) still exhibited gel retraction that began near the gel posts after 2 d (Fig. S2a) and prevented further microvascular network formation, which continued to increase through 6 d (Fig. S2i). The retraction of CGSs are shown from the areas highlighted by the dashed yellow lines (Figs. S2a and S2i). The series of Z-stack images with the step size of 3.5 µm and cross-sectional image with XZ and YZ axis of the area highlighted by the dashed red line in Fig. S2b, that is higher magnification image of the area highlighted by the dashed red line in Fig. S2a, are shown in Figs. S2c–S2g and Fig. S2h, respectively. We noticed no lumen structure at day 2. (Fig. S2h) and insufficient lumen structure at day 6 (Fig. S2i), indicated by the red arrows, with serious gel retraction in the areas highlighted by the dashed yellow lines at day 6 (Fig. S2i).

**
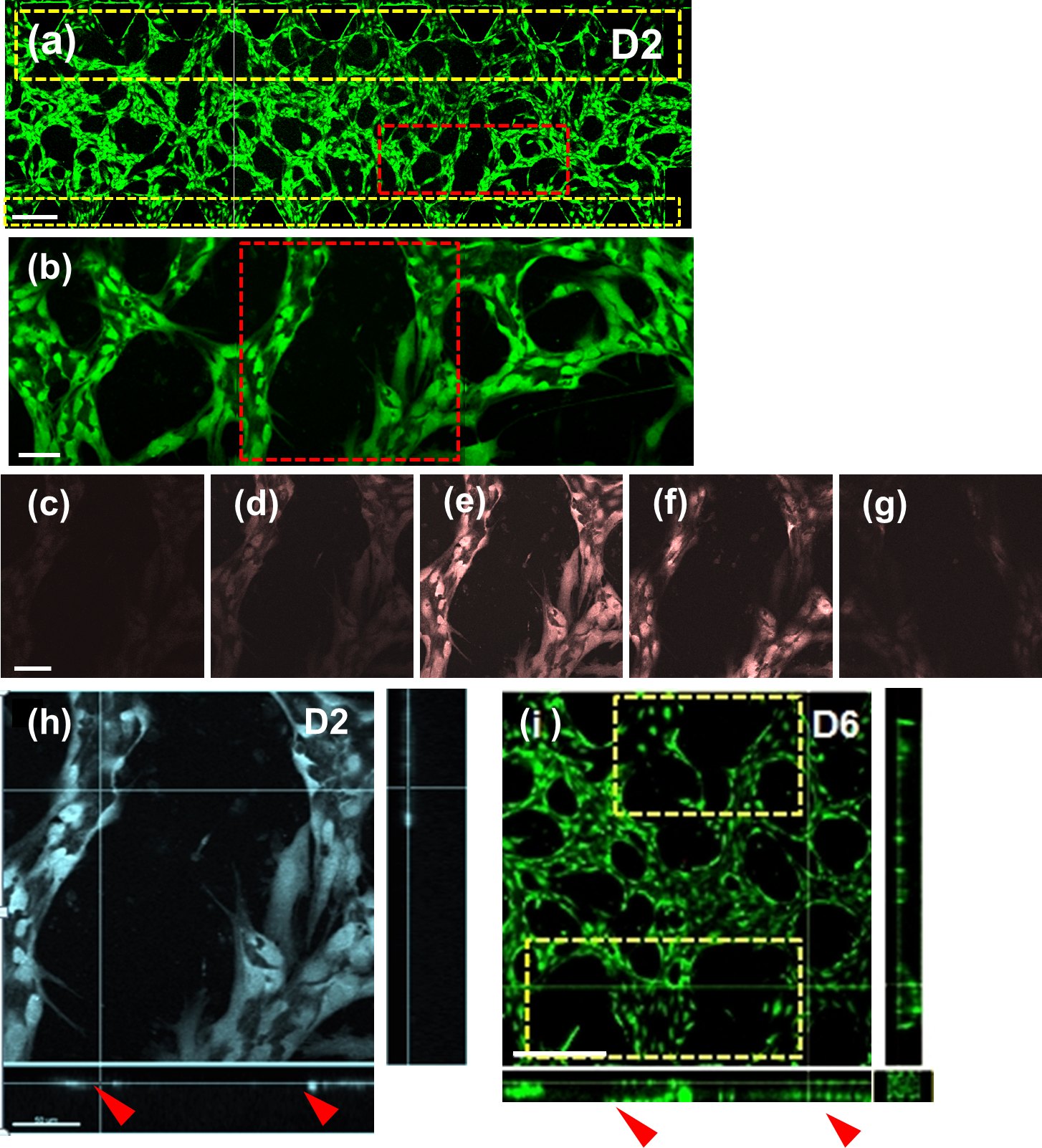
**

**FIGURE S2. Characterization of stabilized microvascular networks within 3D CGSs at day 2 (D2) after initial seeding of HUVECs cultured with GM6001. The concentration of type-I collagen gel was adjusted to 2.5 mg/ml. (a) Representative live confocal images of microvascular networks fully stabilized within 3D CGSs. The areas highlighted by the dashed yellow lines show the retraction of CGSs. Scale bar, 300 µm. (b) Higher magnification image of the area highlighted by the dashed red line in (a). Scale bar, 50 µm. (c)–(g) Series of Z-stack images of the area highlighted by the dashed red line in (b) with the step size of 3.5 µm. Scale bar, 50 µm. (h) Cross-sectional image of the area highlighted by the dashed red line in (b) with XZ and YZ axis. The red arrows indicate no existence of lumen structures. Scale bar, 50 µm. (i) Characterization of stabilized microvascular networks within 3D CGSs at day 6 (D6) after initial seeding of HUVECs cultured with GM6001. The concentration of type-I collagen gel was adjusted to 2.5 mg/ml. The areas highlighted by the dashed yellow lines show the retraction of CGSs. Cross-sectional images by XZ and YZ axis represent insufficient lumen formation within 3D CGSs. The red arrows indicate insufficient lumen structures. Scale bar, 300 µm.**

**References**

^1^ Fishman, E. K., D. R. Ney, D. G. Heath, F. M. Corl, K. M. Horton and P. T. Johnson. Volume rendering versus maximum intensity projection in CT angiography/What works best, when, and why. *RadioGraphics.* 26:905–922, 2006.

^2^ Kirbas, C. and F. Quek. A review of vessel extraction techniques and algorithms. *ACM Comput. Surv.* 36: 81–12, 2004.

^3^ Rashband, W., Image J, Bethesda, Maryland. U. S. *National Institutes of Health*. 1997–2009.

^4^ Soyer, P., D. Heath, D. A. Bluemke, M. A. [Choti](http://www.ncbi.nlm.nih.gov/pubmed?term=Choti%20MA%5BAuthor%5D&cauthor=true&cauthor_uid=8576462), J. E. [Kuhlman](http://www.ncbi.nlm.nih.gov/pubmed?term=Kuhlman%20JE%5BAuthor%5D&cauthor=true&cauthor_uid=8576462), R. [Reichle](http://www.ncbi.nlm.nih.gov/pubmed?term=Reichle%20R%5BAuthor%5D&cauthor=true&cauthor_uid=8576462) and E. K. [Fishman](http://www.ncbi.nlm.nih.gov/pubmed?term=Fishman%20EK%5BAuthor%5D&cauthor=true&cauthor_uid=8576462). Three-dimensional helical CT of intrahepatic venous structures/comparison of three rendering techniques. *J. Comput. Assist. Tomogr.* 20:122–127, 1996.

^5^ Nakayama, Y., M. Imuta, Y. Funama, M. [Kadota](http://www.ncbi.nlm.nih.gov/pubmed?term=Kadota%20M%5BAuthor%5D&cauthor=true&cauthor_uid=12553340), D. [Utsunomiya](http://www.ncbi.nlm.nih.gov/pubmed?term=Utsunomiya%20D%5BAuthor%5D&cauthor=true&cauthor_uid=12553340), S. [Shiraishi](http://www.ncbi.nlm.nih.gov/pubmed?term=Shiraishi%20S%5BAuthor%5D&cauthor=true&cauthor_uid=12553340), Y. [Hayashida](http://www.ncbi.nlm.nih.gov/pubmed?term=Hayashida%20Y%5BAuthor%5D&cauthor=true&cauthor_uid=12553340) and Y. [Yamashita](http://www.ncbi.nlm.nih.gov/pubmed?term=Yamashita%20Y%5BAuthor%5D&cauthor=true&cauthor_uid=12553340). CT portography by multidetector helical CT/comparison of three rendering models. *Radiat. Med.* 20: 273–279, 2002.

^6^ Rubin, G. D., M. D. Dake, S. Napel, C. H. [McDonnell](http://www.ncbi.nlm.nih.gov/pubmed?term=McDonnell%20CH%5BAuthor%5D&cauthor=true&cauthor_uid=8259402), F. G. [Sommer](http://www.ncbi.nlm.nih.gov/pubmed?term=Sommer%20FG%5BAuthor%5D&cauthor=true&cauthor_uid=8259402), L. [Wexler](http://www.ncbi.nlm.nih.gov/pubmed?term=Wexler%20L%5BAuthor%5D&cauthor=true&cauthor_uid=8259402) and D. M. [Williams](http://www.ncbi.nlm.nih.gov/pubmed?term=Williams%20DM%5BAuthor%5D&cauthor=true&cauthor_uid=8259402). Spiral CT of renal artery stenosis/comparison of three-dimensional rendering techniques. *Radiology.* 190:181–189, 1994.

^7^ Achenbach, S., D. Ropers, M. Regenfus, G. Muschiol, W. G. Daniel and W. Moshage. Contrast enhanced electron beam computed tomography to analyse the coronary arteries in patients after acute myocardial infarction. *Heart.* 84:489–493, 2000.

^8^ Calhoun, P. S., B. S. Kuszyk, D. G. Heath, J. C. Carley and E. K. Fishman. Three-dimensional volume rendering of spiral CT data/theory and method. *RadioGraphics.* 19:745–764, 1999.

^9^ Leclerc, X., O. Godefroy, C. Lucas, J. F. [Benhaim](http://www.ncbi.nlm.nih.gov/pubmed?term=Benhaim%20JF%5BAuthor%5D&cauthor=true&cauthor_uid=10207466), T. S. [Michel](http://www.ncbi.nlm.nih.gov/pubmed?term=Michel%20TS%5BAuthor%5D&cauthor=true&cauthor_uid=10207466), D. [Leys](http://www.ncbi.nlm.nih.gov/pubmed?term=Leys%20D%5BAuthor%5D&cauthor=true&cauthor_uid=10207466) and J. P. [Pruvo](http://www.ncbi.nlm.nih.gov/pubmed?term=Pruvo%20JP%5BAuthor%5D&cauthor=true&cauthor_uid=10207466). Internal carotid arterial stenosis/CT angiography with volume rendering. *Radiology.* 210:673–682, 1999.

^10^Ofer, A., S. S. Nitecki, S. Linn, M. [Epelman](http://www.ncbi.nlm.nih.gov/pubmed?term=Epelman%20M%5BAuthor%5D&cauthor=true&cauthor_uid=12591682), D. [Fischer](http://www.ncbi.nlm.nih.gov/pubmed?term=Fischer%20D%5BAuthor%5D&cauthor=true&cauthor_uid=12591682), T. [Karram](http://www.ncbi.nlm.nih.gov/pubmed?term=Karram%20T%5BAuthor%5D&cauthor=true&cauthor_uid=12591682), D. [Litmanovich](http://www.ncbi.nlm.nih.gov/pubmed?term=Litmanovich%20D%5BAuthor%5D&cauthor=true&cauthor_uid=12591682), H. [Schwartz](http://www.ncbi.nlm.nih.gov/pubmed?term=Schwartz%20H%5BAuthor%5D&cauthor=true&cauthor_uid=12591682), A. [Hoffman](http://www.ncbi.nlm.nih.gov/pubmed?term=Hoffman%20A%5BAuthor%5D&cauthor=true&cauthor_uid=12591682) and A. [Engel](http://www.ncbi.nlm.nih.gov/pubmed?term=Engel%20A%5BAuthor%5D&cauthor=true&cauthor_uid=12591682). Multidetector CT angiography of peripheral vascular disease: a prospective comparison with intraarterial digital subtraction angiography. *Am. J. Roentgenol.* 180:719–724, 2003.

^11^Kim, J. K., J. H. Kim, S. J. Bae and K. S. Cho. CT angiography for evaluation of living renal donors: comparison of four reconstruction methods. *Am. J. Roentgenol.* 183:471–477, 2004.

^12^Johnson, P. T., D. G. Heath, B. S. Kuszyk and E. K. Fishman. CT angiography with volume rendering/ advantages and applications in splanchnic vascular imaging. *Radiology.* 200: 564–568, 1996.

^13^Urban, B. A., L. E. Ratner and E. K. Fishman. Three-dimensional volume-rendered CT angiography of the renal arteries and veins/normal anatomy, variants and clinical applications. *RadioGraphics.* 21:373–386, 2001.

^14^Schreiner, S., C. B. Paschal and R. L. Galloway. Comparison of projection algorithms used for the construction of maximum intensity projection images. *J. Comput. Assist. Tomogr.* 20:56–67, 1996.

^15^Dalrymple, N. C., S. R. Prasad, M. W. Freckleton and K. N. Chintapalli. Informatics in radiology (info-RAD): introduction to the language of three-dimensional imaging with multidetector CT. *RadioGraphics.* 25:1409–1428, 2005.
